# Pyrrolo [3,4-*b*]-quinolin-9-amine compound FZU-0038-056 suppresses triple-negative breast cancer partially through inhibiting the expression of Bcl-2

**DOI:** 10.18632/aging.103232

**Published:** 2020-05-23

**Authors:** Danping Wang, Zhi Nie, Xiaoyan Jiang, Jinxiang Ye, Zhimin Wei, Dating Cheng, Chenyang Wang, Yingying Wu, Rong Liu, Haijun Chen, Ceshi Chen, Chunyan Wang

**Affiliations:** 1 Department of Pathology, First Affiliated Hospital of Kunming Medical University, Kunming 650032, Yunnan, China; 2 Department of Neurology, First Affiliated Hospital of Kunming Medical University, Kunming 650032, Yunnan,, China; 3 Key Laboratory of Animal Models and Human Disease Mechanisms of the Chinese Academy of Sciences and Yunnan Province, Kunming Institute of Zoology, Chinese Academy of Sciences, Kunming 650223, China; 4 Kunming College of Life Science, University of the Chinese Academy of Sciences, Kunming 650204, China; 5 Medical Faculty of Kunming University of Science and Technology, Kunming 650500, China; 6 College of Chemistry, Fuzhou University, Fuzhou 350116, Fujian, China; 7 Department of Pathology, The Affiliated Hospital of Qingdao University, Qingdao 266071, China; 8 Translational Cancer Research Center, Peking University First Hospital, Beijing 100034, China; 9 KIZ-CUHK Joint Laboratory of Bioresources and Molecular Research in Common Diseases, Kunming Institute of Zoology, Chinese Academy of Sciences, Kunming 650223, China; 10 Institute of Translation Medicine, Shenzhen Second People’s Hospital, The First Affiliated Hospital of Shenzhen University, Shenzhen 518035, China

**Keywords:** triple-negative breast cancer, tetrahydro-β-carboline derivatives, apoptosis, Bcl-2

## Abstract

Triple-negative breast cancer (TNBC) has a poorer prognosis than other subtypes of breast cancer; however, it lacks effective targeted therapies clinically. In this study, we found FZU-0038-056, a novel compound derived from last-stage functionalization of tetrahydro-β-carboline scaffold, showed the most potent anti-cancer activity against TNBC cells among the 42 synthesized derivatives. We found FZU-0038-056 significantly induces apoptosis in HCC1806 and HCC1937 TNBC cells. FZU-0038-056 reduces the expression levels of several anti-apoptosis proteins, including Bcl-2, Mcl-1 and XIAP. Furthermore, we found FZU-0038-056 induces apoptosis partially through inhibiting the expression of Bcl-2. Finally, we found FZU-0038-056 significantly suppresses HCC1806 xenograft tumor growth in nude mice without affecting their body weight. Therefore, FZU-0038-056 has the potential to be a new anticancer agent for treating human TNBC.

## INTRODUCTION

Breast cancer ranks first among female malignant tumors [1]. According to the cancer statistics of China, the incidence of breast cancer in China is increasing at a rate of 3–4 % per year, and the age of onset is also gradually becoming younger [2]. Based on gene expression profiles, breast cancer can be divided into four subtypes, luminal A, luminal B, HER2 positive, and basal-like/triple-negative breast cancers (TNBC) [3, 4]. Among all the subtypes of breast cancer, TNBC is more aggressive and has higher rates of relapse and metastasis than other subtypes. In the past decades, hormone therapy and anti-HER2 targeted therapy have significantly improved the prognosis of ERα/PR+ and HER2+ breast cancers, respectively. However, TNBC does not have effective targeted therapies for lacking the expression of ERα, PR and HER2, chemotherapy remains the major option for TNBC patients [5]. Therefore, it is important to develop effective treatments for TNBC.

Mitochondria are cellular organelles that engage in aerobic respiration. In addition to producing ATP, they are also involved in apoptosis. Mitochondria are the central organelle in the intrinsic apoptotic pathway, acting through the release of cytochrome C, Smac/DIABLO (direct IAP binding protein) and apoptosis-inducing factor AIF, while Bcl-2 can inhibit apoptosis by inhibiting the release of cytochrome C and preventing the translocation of Smac/DIABLO and the apoptosis-inducing factor AIF [6, 7].

The tetrahydro-β-carboline (THβC) skeleton as one important class of natural indole alkaloids commonly was found in nature. It is a very important skeleton and is present in many pharmacologically active alkaloids such as reserpine, tadalafil, and tetrahydro-halamine [8]. In recent years, tetrahydro-β-carboline derivatives have also become popular in terms of cancer targeted therapy. The TGF-β signaling pathway [9], phosphodiesterase (PDE) [10, 11], and spindle kinesin (KSP) [12, 13] are potential anti-tumor targets of tetrahydro-β-carboline derivatives. To develop more potent anticancer reagents, we designed and synthesized 42 pyrrolo [3,4-*b*]-quinolin-9-amines by using THβC as the starting point through an oxidative rearrangement coupling reaction.

There are no reports on the anti-tumor activity of pyrrolo [3,4-*b*]-quinolin-9-amine compounds to date. In this study, we screened anti-cancer activities of the 42 compounds and identified FZU-0038-056 to be the most potent one. FZU-0038-056 significantly induced apoptosis in HCC1806 and HCC1937. We showed that FZU-0038-056 reduced the protein expression levels of Bcl-2, Mcl-1 and XIAP. Bcl-2 overexpression partially reduced the pro-apoptosis of FZU-0038-056. These findings suggest that FZU-0038-056 is a mitochondrial apoptotic pathway activator and may be an effective anticancer agent for the treatment of TNBC.

## RESULTS

### FZU-0038-056 is the most potent anti-cancer compound among the 42 tested tetrahydro-β-carboline derivatives

**Figure 1:**
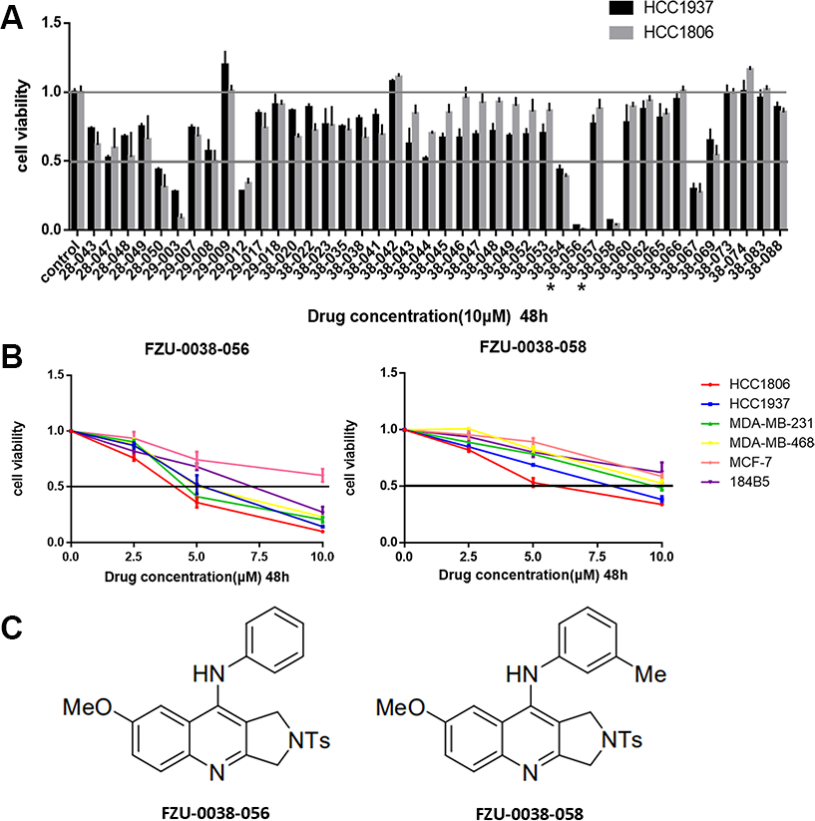
**Identification of FZU-0038-056 as a potent anti-cancer compound in TNBC cell lines.** (**A**) The HCC1806 and HCC1937 breast cancer cell lines were treated with 42 different compounds (10 μM) for 48 hours. DMSO was used as the negative control. Cell viability was measured using SRB assays. FZU-0038-056 and FZU-0038-058 labeled with an asterisk were selected for further studies. (**B**) Four different TNBC cell lines, two ER positive breast cancer cell lines and one human immortalized breast cell line were treated with DMSO control or FZU-0038-056/FZU-0038-058 at indicated concentrations (2.5, 5 and 10 μM, respectively) for 48 hours. Cell viability was measured using SRB assay. (**C**) The chemical structures of FZU-0038-056 and FZU-0038-058.

The pyrrolo [3,4-*b*]-quinolin-9-amine compound is a novel scaffold derived from tetrahydro-β-carboline derivatives [8], and its anti-tumor roles has not been reported yet. We treated two human TNBC cell lines (HCC1806 and HCC1937) with 42 pyrrolo [3,4-*b*]-quinolin-9-amine compounds (10 μM) for 48 hours and the cell viability was measured by SRB assays. Among the 42 compounds, FZU-0038-056 and FZU-0038-058 showed the most potent cytotoxicity against both cancer cell lines (Figure 1A). Then, we examined the half maximal inhibitory concentrations (IC_50_) of FZU-0038-056 and FZU-0038-058 in four TNBC cell lines (HCC1937, HCC1806, MDA-MB-231 and MDA-MB-468), one ERα positive breast cancer cell line (MCF-7) and the human immortalized breast epithelial cell line 184B5. Both FZU-0038-056 and FZU-0038-058 showed stronger cytotoxic effects in TNBC cells than in ERα positive and immortalized cells (Figure 1B). Because the structure of FZU-0038-058 is similar to FZU-0038-056 (Figure 1C), while its inhibitory effect is weaker than that of FZU-0038-056 in breast cancer cells (Figure 1B), we selected compound FZU-0038-056 for further *in vitro* and *in vivo* anticancer studies. The IC_50_s of FZU-0038-056 in the HCC1806 and HCC1937 cells were 3–6 μM (Figure 1B).

### FZU-0038-056 induces apoptosis in HCC1806 and HCC1937 cells

**Figure 2:**
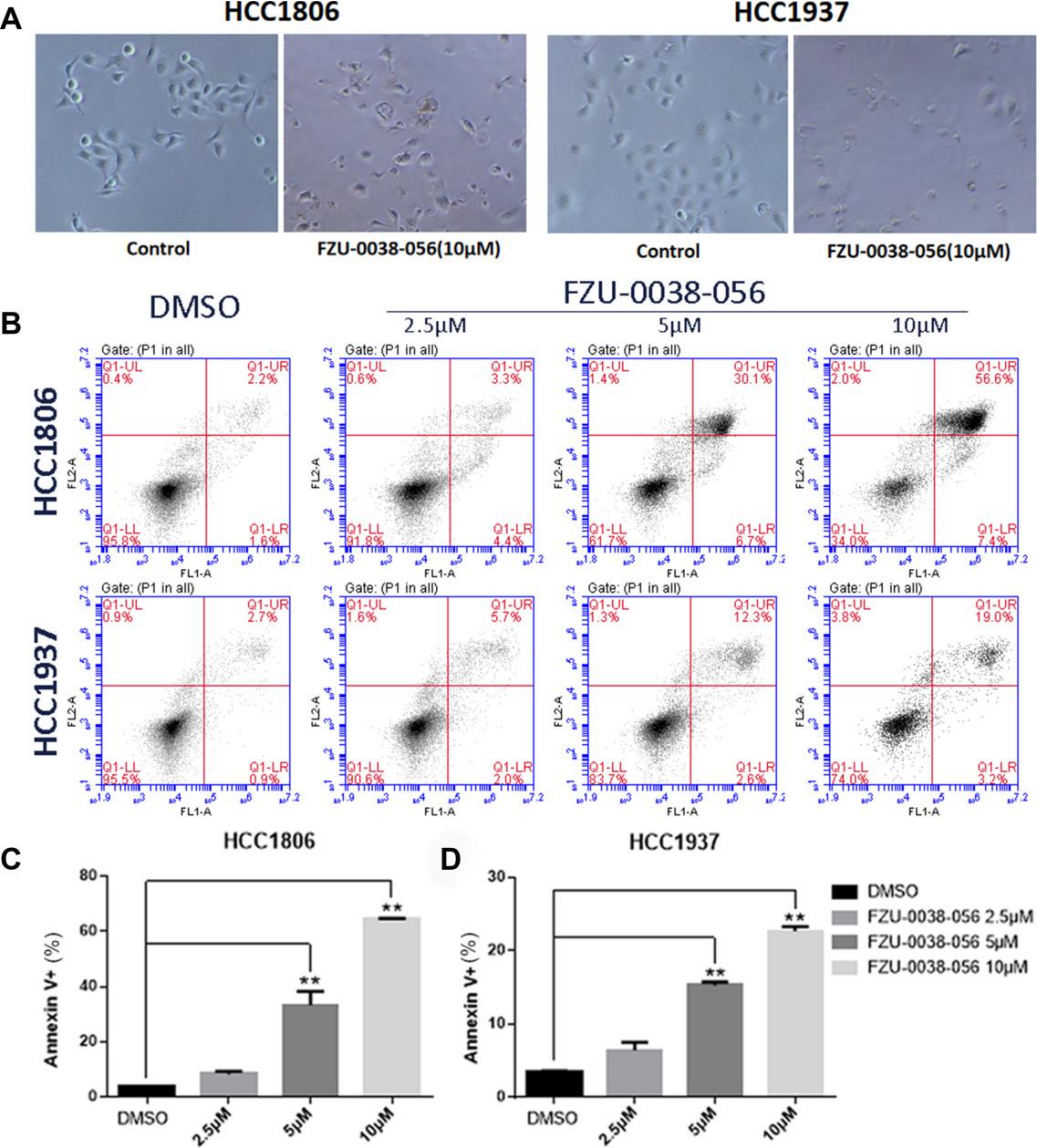
**FZU-0038-056 induces apoptosis in HCC1806 and HCC1937 cells.** (**A**) The cell morphology changes of HCC1806 and HCC1937 cells after the treatment of FZU-0038-056 (10μM) for 12 hours. (**B**) HCC1806 and HCC1937 cells were stained with Annexin V/PI and analyzed by flow cytometry analysis after the cells were treated with FZU-0038-056 (2.5, 5, 10 μM) for 24 hours. DMSO was added as the negative control. (**C**, **D**) The percentages of Annexin V-positive cells from panel B are shown. ** p < 0.01.

Since both cell growth inhibition and apoptosis reduce cell viability, we investigated the effects of FZU-0038-056 on cell growth and apoptosis. After treating with FZU-0038-056 (10 μM) for 12 hours, HCC1806 and HCC1937 cells became round and detached (Figure 2A). To test whether FZU-0038-056 induced apoptosis, we further measured the apoptosis of HCC1806 and HCC1937 cells by Annexin V/PI staining using flow cytometry analysis. FZU-0038-056 (2.5–10 μM) induced apoptosis in both the HCC1806 and HCC1937 cell lines in dose-dependent manners (Figure 2B–2D). We also performed cell cycle analysis in HCC1806 and HCC1937 cells after FZU-0038-056 treatment. However, FZU-0038-056 did not affect cell cycle progression significantly in TNBC cells (Supplementary Figure 1).

### FZU-0038-056 regulates the expression of apoptosis-related genes

**Figure 3:**
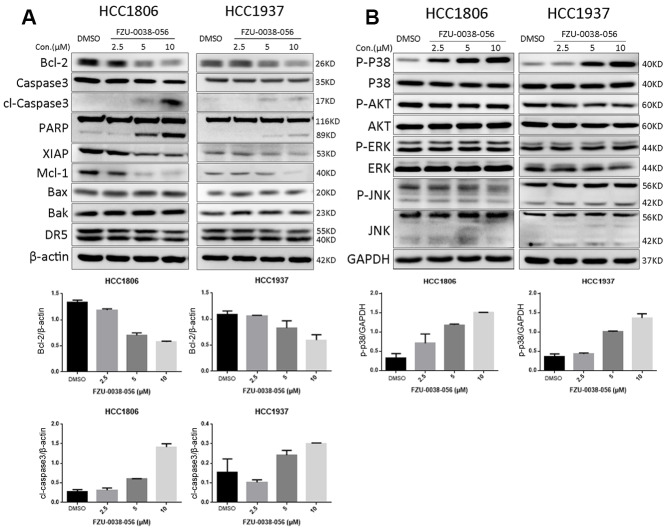
**FZU-0038-056 decreases the expression of anti-apoptosis proteins and increased p38 phosphorylation in HCC1806 and HCC1937 cells.** (**A**) HCC1806 and HCC1937 cells were treated with FZU-0038-056 (2.5, 5, 10 μM) for 24 hours. Cell lysates were collected for immunoblotting to test the protein levels of cleaved Caspase-3, and PARP, Bcl-2, XIAP, Mcl-1; Bax, Bak and DR5. β-actin was used as the loading control. The quantification data of cl-caspase-3 and Bcl-2 protein expression were shown under the immunoblot images. (**B**) HCC1806 and HCC1937 cells were treated with FZU-0038-056 (2.5, 5, 10 μM) for 24 hours. The protein levels of p38, p-p38, AKT, p-AKT, ERK, p-ERK, JNK and p-JNK were examined by WB. GAPDH was used as the loading control. The quantification data of p-p38 protein expression was shown under the immunoblot images.

Since FZU-0038-056 induced apoptosis in HCC1806 and HCC1937 cells, we further examined the expression of apoptosis related genes by WB. FZU-0038-056 treatment increased the cleavage of caspase-3 and PARP in the HCC1806 and HCC1937 cell lines (Figure 3A). Furthermore, it significantly decreased the protein expression levels of several anti-apoptotic proteins, including Bcl-2, XIAP, and Mcl-1, in a dose-dependent manner (Figure 3A). In contrast, we did not observe an increase of expression of pro-apoptosis proteins, including Bax, Bak, and DR5 (Figure 3A).

In addition, we examined the activation of several major apoptosis-related signaling proteins, including p38, JNK, ERK, and AKT. We found FZU-0038-056 increased the phosphorylation level of p38, but not the other tested kinases, in HCC1806 and HCC1937 cells in a dose-dependent manner (Figure 3B).

### FZU-0038-056 does not induce TNBC apoptosis through activating p38

**Figure 4:**
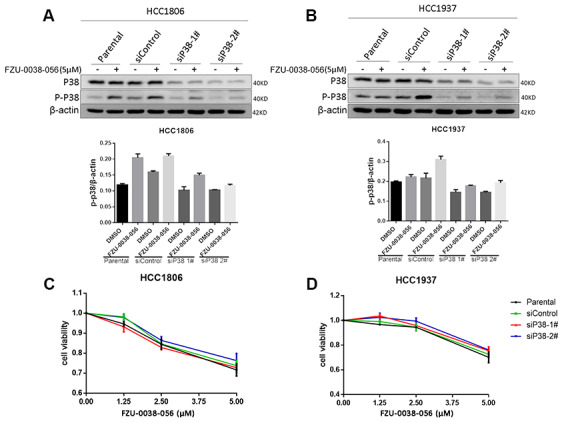
**FZU-0038-056 induces TNBC apoptosis not through activating p38.** (**A, B**) HCC1806 and HCC1937 cells were transfected with siRNAs targeting p38 for 36 hours, and treated with FZU-0038-056 (5 μM) or DMSO for 24 hours. Cell lysates were collected for WB assay. The quantification data of p-p38 protein expression was shown under the immunoblot images. (**C, D**) HCC1806 and HCC1937 cells were transfected with siRNA for 36 hours, then treated with FZU-0038-056 (1.25, 2.5, 5 μM) or DMSO for 48 hours. Cell viability was measured via the SRB assay.

The p38 MAPK signaling pathway is well known to play important roles in various physiological processes, including apoptosis [14]. To test whether p38 activation leads to apoptosis, we knocked down p38 using two different siRNAs in HCC1806 and HCC1937 cells (Figure 4A, 4B). However, depletion of p38 did not attenuate the cell survival inhibition effects of FZU-0038-056 in either of the tested TNBC cell lines.

### FZU-0038-056 induces apoptosis partially through inhibiting the expression of Bcl-2

**Figure 5:**
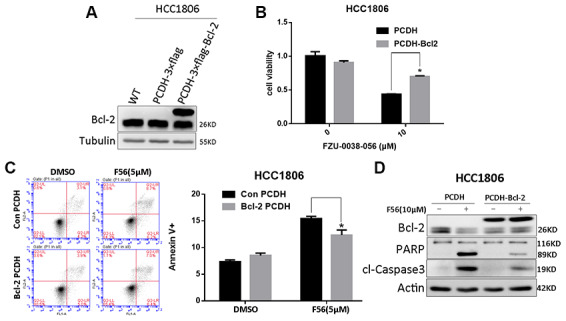
**FZU-0038-056 induced apoptosis partially via inhibiting Bcl-2 in HCC1806 cells.** (**A**) Bcl-2 was overexpressed in HCC1806 cells through a lentivirus system. (**B**) Overexpression of Bcl-2 reduced the cell viability inhibition triggered by FZU-0038-056. Cell viability was measured by the SRB assay. *p < 0.05. (**C**) FZU-0038-056-induced apoptosis were partially rescued by the overexpression of Bcl-2 compared with the control cells, as measured by Annexin V staining and flow cytometry analysis. Annexin V positive cell populations were quantified and shown on the right side. *p < 0.05. (**D**) FZU-0038-056-induced apoptosis were partially rescued by the overexpression of Bcl-2, as measured by Caspase-3 and PARP cleavage. The cells were treated with DMSO or FZU-0038-056 (10 μM) for 24 hours, and cell lysates were collected for WB analysis. β-actin was used as loading control.

Since FZU-0038-056 downregulated the expression of Bcl-2, a key anti-apoptosis protein (Figure 3A), we further examined whether FZU-0038-056 facilitates apoptosis through Bcl-2. We overexpressed Bcl-2 according to published protocols [15] in HCC1806 cells (Figure 5A) and found that FZU-0038-056-induced cell viability loss was significantly rescued (Figure 5B). Moreover, we demonstrated that Bcl-2 overexpression significantly reduced FZU-0038-056-induced apoptosis, as measured by Annexin V staining (Figure 5C). Consistently, FZU-0038-056-induced cleavage of caspase-3 and PARP were also alleviated in Bcl-2 overexpressing HCC1806 cells (Figure 5D).

### FZU-0038-056 suppresses HCC1806 xenograft tumor growth in nude mice

**Figure 6:**
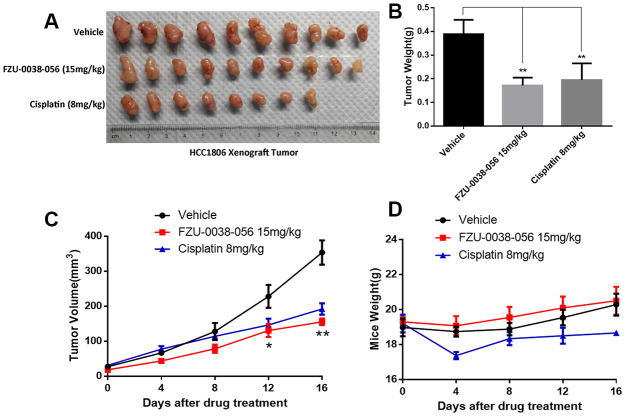
**FZU-0038-056 suppresses HCC1806 xenograft tumor growth in nude mice.** (**A**) FZU-0038-056 suppresses HCC1806 xenograft tumor growth in nude mice. Tumors were collected after the mice were treated with FZU-0038-056, vehicle, or cisplatin for 20 days. (**B**) FZU-0038-056 significantly reduced HCC1806 xenograft weights. Average tumor weights are graphed (n=5). **p < 0.01. (**C**) Tumor growth was significantly suppressed by FZU-0038-056 (15 mg/kg) and cisplatin (8 mg/kg) compared with vehicle control. **p < 0.01, *p < 0.05. (**D**) Compared to vehicle control, FZU-0038-056 (15 mg/kg) did not significantly reduce the mouse body weights although cisplatin (8 mg/kg) reduced the mouse body weights significantly.

To evaluate the antitumor activity of FZU-0038-056 *in vivo*, we established a HCC1806 xenograft model via subcutaneously injecting cancer cells into mammary fad pads of female nude mice. As the data show in Figure 6, tumor growth was significantly suppressed by FZU-0038-056 compared with the negative control. Compared with cisplatin (8mg/kg), FZU-0038-056 (15mg/kg) showed a similar anti-tumor effect but had less impact on the body weights of the mice (Figure 6A–6D).

Although FZU-0038-056 could efficiently kill cancer cells in our study, the dosage used is relatively high *in vitro* (Figure 1A, 1B) and *in vivo* (Figure 6A, 6C). Considering the side effects of cisplatin is severe, while FZU-0038-056 is much safer *in vivo* (Figure 6D), we tried to check whether FZU-0038-056 could strengthen the anti-tumor effect of cisplatin, thus cisplatin could be applied at lower dosages. As the data shown in Supplementary Figure 2, on one hand, cisplatin suppressed triple-negative breast cancer cell HCC1806 survival more efficiently when it was used together with FZU-0038-056. The IC50 of cisplatin was reduced from 6.5 μM to 2.7 μM (Supplementary Figure 2A, 2B) when 5 μM FZU-0038-056 was added. On the other hand, cisplatin also strengthened the anti-cancer activity of FZU-0038-056. The IC50 of FZU-0038-056 was reduced from 6.9 μM to 4.5 μM (Supplementary Figure 2C, 2D) when 5 μM cisplatin was added to treat HCC1806 cells. Taken together, our results show combination of FZU-0038-056 and cisplatin synergistically suppressed HCC1806 cell survival.

## DISCUSSION

In this study, we examined the cytotoxicity of 42 pyrrolo [3,4-*b*]-quinolin-9-amine compounds in two TNBC cell lines and selected the most potent anti-cancer compound FZU-0038-056 for further study. We demonstrated that FZU-0038-056 significantly inhibited TNBC cell survival. FZU-0038-056 induces apoptosis partially by inhibiting Bcl-2 expression. Finally, we demonstrated that FZU-0038-056 significantly suppressed HCC1806 xenograft tumor growth *in vivo* without affecting mouse body weight.

Apoptosis is one of the main mechanisms by which drugs inhibit tumor growth. There are three main pathways for apoptosis: the mitochondrial pathway, the death receptor pathway, and the endoplasmic reticulum pathway [16]. The mitochondrial programmed death pathway is a process in which multiple factors are interrelated and balance each other to promote cell death [17, 18]. When the apoptotic signal is transduced into mitochondria, the mitochondrial outer membrane forms poly channels, and mitochondrial contents such as cytochrome C (cytC), Smac and AIF are released [19]. Smac can relieve the inhibition of precursor caspase by IAPs. AIF induces fragmentation of DNA and concentration of chromosomes [19]. CytC can activate caspase-9 [20]. In this study, FZU-0038-056 downregulated the expression of Bcl-2 and Mcl-1 and induced the cleavage of caspase-3 and PARP (Figure 3). Meanwhile, the proapoptotic effect of FZU-0038-056 on TNBC cells can be significantly reduced by ectopically overexpressed Bcl-2 (Figure 5). Therefore, FZU-0038-056 activates the intrinsic mitochondrial apoptosis pathway partially by inhibiting the expression of Bcl-2. However, the exact mechanism by which FZU-0038-056 inhibits the expression of Bcl-2 is still unclear and requires further investigation. It would be interesting to identify direct binding molecules of FZU-0038-056.

Additionally, we found that FZU-0038-056 activated the p38 MAPK (Figures 3B and 4). However, when we depleted p38 expression using siRNAs, FZU-0038-056-induced apoptosis was not blocked (Figure 4), indicating that FZU-0038-056 does not induce apoptosis via activating the p38 pathway.

In summary, we demonstrated that FZU-0038-056 induces apoptosis of TNBC cells and inhibits TNBC tumor growth in nude mice. The mechanism by which FZU-0038-056 inhibits TNBC may involve the suppression of Bcl-2. Interestingly, we found combination application of FZU-0038-056 and cisplatin synergistically suppressed HCC1806 cell survival, which implicates possible application of FZU-0038-056 in combination with clinically used anti-tumor agents, including cisplatin. Therefore, FZU-0038-056 has potential as a novel anticancer agent for human TNBC.

## MATERIALS AND METHODS

### Compounds and cell lines

FZU-0038-056 and other compounds were designed and synthesized from THβC as the lead compound [8]. FZU-0038-056 was dissolved in DMSO and diluted in the corresponding culture media for the experiments. All of the cell lines used in this study were purchased from the American Type Culture Collection (ATCC) and validated by STR analysis (Kunming Cell Bank, Kunming Institute of Zoology, Chinese Academy of Sciences). HCC1806 and HCC1937 were cultured in RPMI-1640 medium supplemented with 10 % fetal bovin serum (FBS). MDA-MB-231 and MDA-MB-468 were cultured in Dulbecco’s Modified Eagle’s Medium (DMEM) with 10 % FBS. MCF7 was cultured in Minimum Essential Medium (MEM) with 10 % FBS and 0.01 mg/ml human recombinant insulin. 184B5 cells were cultured in DMEM/F12 with 10 % FBS, 5 μg/ml insulin and 10 ng/ml cholera toxin. All cells were maintained at 37 °C with 5 % CO_2_ in a humidified atmosphere.

### Antibodies

The anti- PARP, XIAP, Mcl-1, Bcl-2, Bax, Bak, DR5, p-JNK, JNK, p38, p-p38, ERK, p-ERK, AKT, p-AKT, and tubulin antibodies were purchased from Cell Signaling (Danvers, MA). The anti-caspase-3 and cleaved caspase-3 antibodies were purchased from Imagenex (San Diego, CA). The anti-β-actin antibody was purchased from Sigma (St. Louis, MO).

### *In vitro* cytotoxicity assays

Normally cultured logarithmic growth phase cells were uniformly seeded at 30,000/well into 48-well plates. The day after plating, the cells were treated with the drugs at the indicated concentrations. Forty-eight hours later, the cells were fixed with 200 μl 10 % TCA (trichloroacetic acid) solution for 1 hour at room temperature, washed 5 times with deionized water, and then dried at room temperature. After drying, the cells were stained with 100 μl of 0.4 % (W/V) SRB in 1 % acetic acid for 5–15 min, followed by washing 5 times with 1 % glacial acetic acid and dried. Finally, 200 μl of 10 mM Tris base solution per well was added, and optical densities were measured at 530 nm in a spectrophotometric plate reader.

### Apoptosis analysis

HCC1806 and HCC1937 were treated with different concentrations of FZU-0038-056 for 24 hours. DMSO was used as the negative control. The cells were stained with Annexin-V (BD, San Diego, CA) for 30 min and PI (BD, San Diego, CA) for 5 min in the dark at room temperature. Finally, the cells were analyzed by flow cytometry.

### Western blotting (WB)

FZU-0038-056 or DMSO treated cancer cells were harvested using lysis buffer supplemented with a protease inhibitor cocktail (Roche Applied Science, Mannheim, Germany) for 30 min on ice. Proteins were collected and centrifuged at 4 °C, 13000 rpm for 10 min. Equal amounts of protein samples were then separated by SDS-PAGE electrophoresis and transferred to polyvinylidene fluoride (PVDF) membranes (Millipore, Bedford, MA). The membrane was blocked in 5 % skim milk for 1 hour, incubated with the primary antibody (1000 × dilution) overnight at 4 °C, washed 3 times with 1 × PBST for 10 min/time, and then incubated with secondary antibodies conjugated with horseradish peroxidase (HRP) (Jackson ImmunoResearch Laboratory, West Grove, PA) for 1 hour. They were then washed 3 times again with 1 × PBST. Finally, the membranes were incubated with Western Lighting Chemiluminescence Reagent Plus (PerkinElmer Life Sciences, Shelton, CT) and images were taken using an ImageQuant LAS4000 Biomolecular imager (GE Healthcare, UK).

### Tumorigenicity assays

Eighteen six-week-old female nude mice were purchased from Hunan SJA Laboratory Animal Co., Ltd., (Changsha, Hunan, China) and were housed in an SPF animal facility. HCC1806 cells (1×10^6^ cells per mouse, resuspended in 75 μl 1×PBS with 20 % Matrigel) were injected subcutaneously near the third mammary fat pad of the mice. When the tumor grew to 50 mm^3^, the mice were randomly divided into 3 groups (n=6). All mice were injected intraperitoneally (i.p.) with FZU-0038-056 solution or control every two days. The negative control group was treated with vehicle solution (5 % DMSO + 95 % saline), the experimental group was treated with FZU-0038-056 (15 mg/kg), and the positive control group was treated with cisplatin (8 mg/kg). The body weights and tumor volumes were measured every 4 days and the tumor volumes were calculated according to the formula V = 0.5 × L × W^2^, where L = length (mm) and W = width (mm). Animal studies were approved by the Institutional Ethics Committee of the Kunming Institute of Zoology, Chinese Academy of Sciences.

### Statistical analysis

All data in this study were analyzed by SPSS 7.0 and are presented as the mean ± SD. Differences between groups were identified using Student’s t-test, and p<0.05 was considered statistically significant.

## Supplementary Material

Supplementary Figures
