# Supplementary material for: Pyrrolo [3,4-b]-quinolin-9-amine compound FZU-0038-056 suppresses triple-negative breast cancer partially through inhibiting the expression of Bcl-2
Source: Aging (Albany NY). 2020 May 23;12(10):9621–32. doi: 10.18632/aging.103232 (PMC7288966; doi:10.18632/aging.103232)
Supplement: Supplementary Figures [file aging-12-103232-s001..pdf]

SUPPLEMENTARY FIGURES

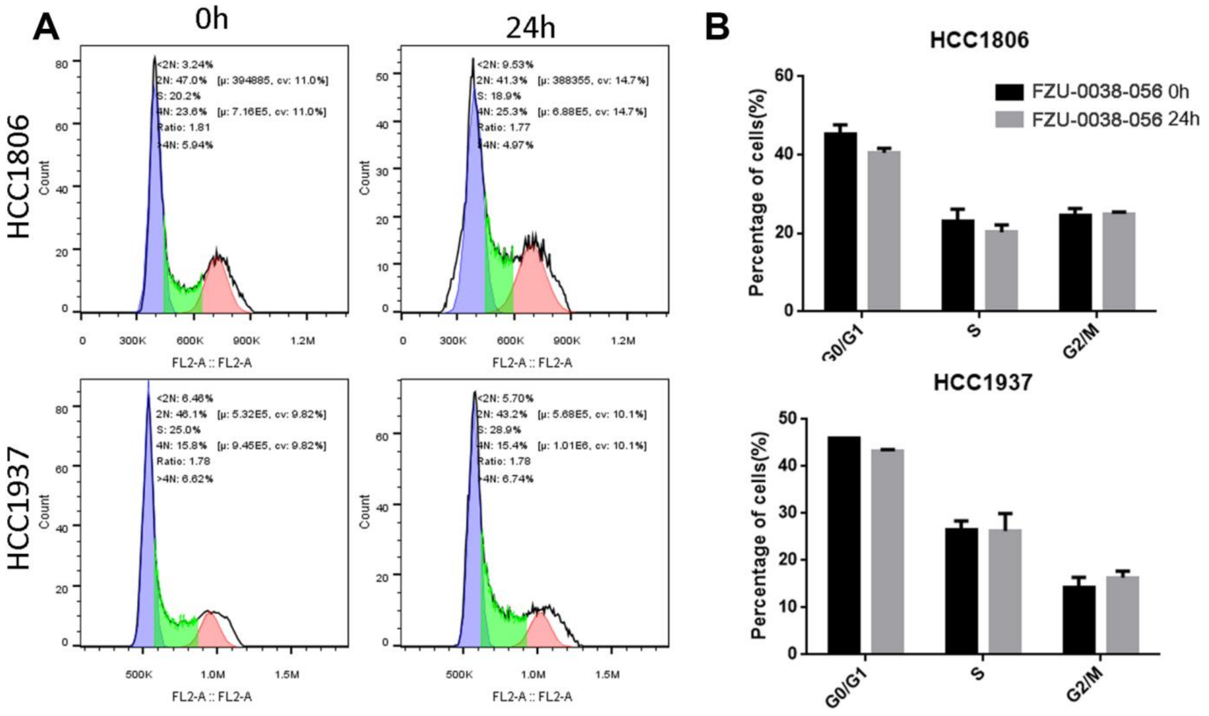

Supplementary Figure 1. FZU-0038-056 does not affect TNBC cell cycle progression. (**A**) The HCC1806 and HCC1937 breast cancer cells were treated with FZU-003-056 (5 µM) for 24 hours. DMSO was used as the negative control. Cells were collected for PI (Propidium Iodide) staining and cell cycle analysis after treatment. (**B**) Quantification of panel A.

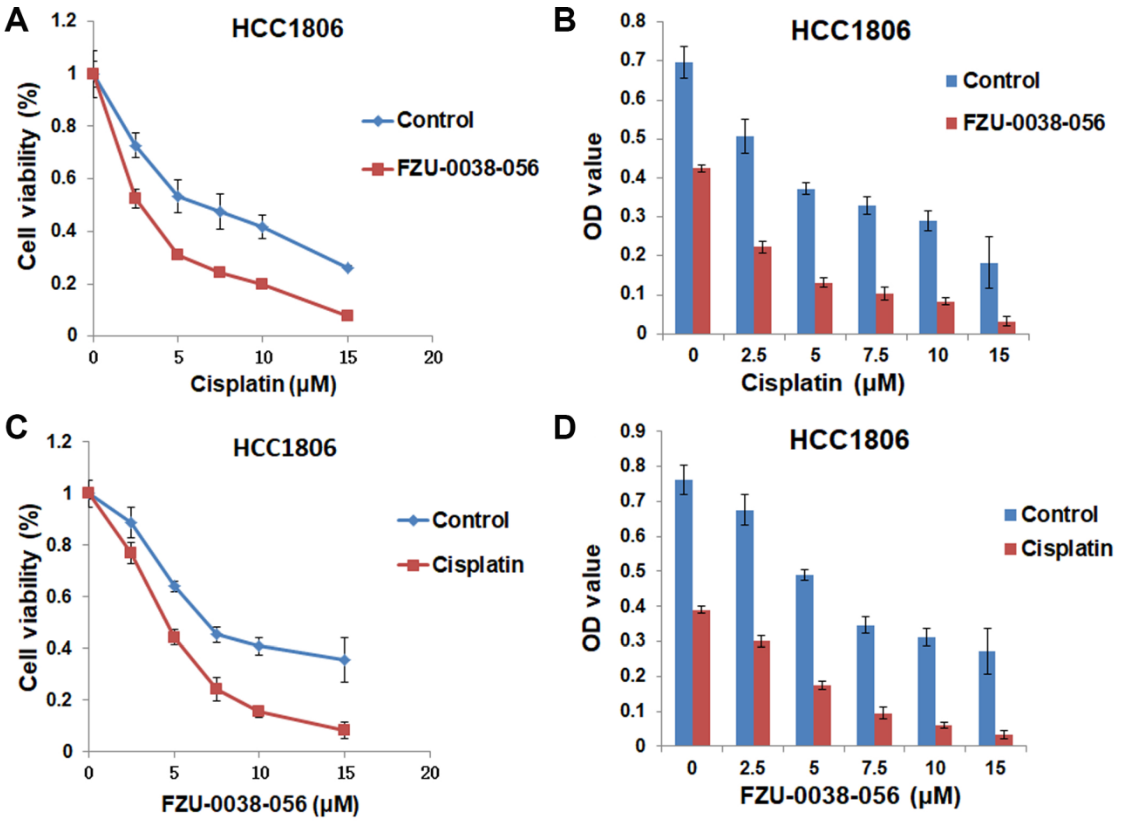

Supplementary Figure 2. FZU-0038-056 and cisplatin synergistically suppress HCC1806 cell survival. (**A**, **B**) HCC1806 cells were treated with FZU-0038-056 (5μM) together with cisplatin at indicated dosages for 48 hours. Cells were fixed for SRB analysis after treatment. (**C**, **D**) HCC1806 cells were treated with cisplatin (5μM) together with FZU-0038-056 at indicated dosages for 48 hours. Cells were fixed for SRB analysis after treatment.
